# Mortality from Eruptive Fevers at Different Periods of the Year

**Published:** 1857-07

**Authors:** J. W. Tripe


TRANSACTIONS

OF THE

EPIDEMIOLOGICAL SOCIETY OF LONDON.

### Papers and Communications.

### THE MORTALITY FROM THE ERUPTIVE FEVERS AT DIFFERENT PERIODS OF THE YEAR.

By J. W. TRIPE, M.D.

A KNOWLEDGE of the period of the year at which the eruptive diseases prove most fatal, and of that at which they cause their minimum of mortality, appears to me an important step towards ascertaining some of the conditions which favour or diminish their spread. In order to obtain this knowledge with as much exactitude as possible, I have formed two sets of tables from the immense mine of information contained in the weekly and other returns of the Registrar-General, and have extended the inquiry over seventeen years, viz., 1840 to 1856 inclusive. For the convenience of future reference, I have divided this period into two; the first containing the ten years, 1840-49, and the other the seven years, 1850-56. During the latter period I have tabulated the return for each week in each year separately; whilst, during the former, I have tabulated only the total of the corresponding weeks in the ten years. In this way I have been able to ascertain the week in which each of these diseases culminated, and *vice versâ*, and how far these events corresponded in each year, and to point out some of the causes of their variations. But the immense labour that would have been necessary fully to consider this subject, being far greater than I could undertake, and the space it would occupy being far in excess of the ordinary limit of a paper, compel me to leave this part of the subject comparatively untouched.

Until the causes which induce death in this country had been registered with some exactitude for many years, no certain data could be obtained for such an investigation as I propose; nor indeed has sufficient time elapsed even now, to place this matter on a firm basis, in consequence of a variation every year in the tendency of all diseases to assume more or less of a certain type, inclining either towards a sthenic or an asthenic form.

As the type of disease, that is to say, its tendency to assume a certain form, varies according to the atmospheric constitution of the year (Sydenham's expression), it is very evident that, if this tendency do not depend on thermal or other seasonal variations, the course of the diseases themselves must be influenced in an uncertain degree by atmospheric variations; for the same results can only be produced by identical causes. The asthenic type which all diseases have had a tendency to assume during the last fifteen years, cannot depend on the thermal variations of the year, as the climate of London has passed through an ascending course of temperature during the years 1841-47, a descending course during the years 1847-52, a sharp ascent in the following year, and a sharp descent in 1854. Further, observations since 1771, registered with great accuracy, which have been subsequently reduced and corrected with great care by Mr. Glaisher, show that the climate of London has passed through a series of ascending and descending curves. I give the highest and the lowest elevations: in 1771 the mean temperature was 45·4°; in 1779, 51·2°; in 1784, 45·1° (1st period); in 1794, 48·9°; in 1797, 45·7° (2nd period); in 1806, 50·5°; in 1814, 45·8° (3rd period); in 1822, 51·0°; in 1829, 46·0° (4th period); in 1834, 51·0°; in 1838, 46·4° (5th period); in 1846, 51·3°; in 1855, 46·6°. We thus see that the thermal line has described a series of ascending and descending lines—not absolutely curves, for the yearly rate of increase or decrease has not been precisely accurate; and yet the type of disease has not followed these thermal elevations and depressions, but has gradually changed, it may be with the habits of the people, but more probably, as similar transitions have been noticed by the older physicians, from other definite but varying causes with which at present we are unacquainted.

In making these observations, it must not be supposed that I ignore the influence of other important atmospheric elements, viz., variations in the humidity, electricity, and weight of the atmosphere, the direction and force of the wind; for I have long since shown that they exercise great power over the mortality of one of the eruptive fevers, viz., scarlet fever. But, as before stated, the difficulty of ascertaining the effects produced on health by a varying intensity of these meteorological elements, and the immense space required for their consideration, prevent me from doing more than allude to the subject. I shall, therefore, confine myself to pointing out the periods at which the eruptive fevers induce their greatest and lowest rates of death, and mention the average temperature of those periods. I shall also show, by a comparison of the deaths in each of the four quarters of the year, and of the mortality in the period 1840-49 with that in 1850-56, and also of the mortality in these quarters with their temperature, that temperature does exercise a great influence on the mortality from the eruptive fevers.

It is not my intention to point out the causes of death in the eruptive diseases, although it is very evident that in classifying deaths under these heads, we are including those from almost all the acute maladies. Thus, measles induce death chiefly from inflammatory disease of the lungs; scarlatina from blood-poisoning, throat disease, inflammations of the solid viscera, etc. The necessity of the case requires that the deaths should be registered on the present plan; hence it is impossible, even if desirable, to separate the uncomplicated from complicated cases. If the inflammatory complications which arise during the progress of cases, whether fatal or otherwise, of the eruptive fevers, could be ascertained in large numbers, and over a long period of years, and then compared with variations in the meteorological elements, much curious and practical information might be obtained.

I have entered the deaths in the week during which they were registered, and not subsequently, as they are usually registered about three days after death; a whole week's alteration would have been too great. The dates are therefore wrong to the extent of three or four days: a space of time too small to deserve much notice.

I. Small-Pox. In the period 1840-49 the mortality from this disease presented two periods of elevation and of depression. The first period of elevation occurred during the first and third weeks, and the second during the twenty-third and twenty-sixth weeks; and those of depression during the fifteenth and thirty-eighth weeks. In the years 1850-56, there were also two periods of elevation and depression: the former occurred in the first, fourth, and fifth weeks, and also in the twentieth and twenty-first weeks: whilst the periods of depression happened in the thirteenth and thirty-ninth weeks. The first period of elevation occurred, therefore, in January in 1840-49, and in January and the first week in February in 1850-56: the second period of highest mortality happened in June in 1840-49 and in May 1850-56. The first period of lowest mortality happened early in April during the years 1840-49, and at the end of March in the years 1850-56: the second period of lowest mortality occurred in the month of September in the periods 1840-49 and 1850-56. During the period included in the years 1840-49, the highest mortality from small-pox in the ten corresponding weeks was 262, which occurred in the first week of the year; in the second week it was 214, in the third 254, and thence it declined to 123 in the fifteenth; it then ascended to 186 in the twenty-sixth week, and again descended to 142 in the thirtieth week, from which it rapidly increased, and remained almost stationary during the forty-fourth to the fifty-first weeks. During the years 1850-56 there were registered in the following seven corresponding weeks these numbers of deaths; in the first week 128, in each of the fourth and fifth weeks 129, in each of the sixth and seventh weeks 128, thence they declined to 83 in the thirteenth week, then increased to 133 in the twentieth week, declined again to 63 in the thirty-ninth week, and gradually rose until, in the last week in the year, the mortality was 117.

In the years 1840-56 the aggregate mortality from small-pox was 14,257. Of this number 3267 deaths were registered in the spring quarter, 3406 in the summer quarter, 3322 in the autumn, and 4262 in the winter. By spring quarter I mean the months of March, April, and May; by the summer, June, July, and August; by autumn, September, October, and November; and by winter, December, January, and February. Of the aggregate mortality, 22·9 per cent. occurred in the spring quarter, 23·9 per cent. in summer, 23·3 in autumn, and 29·9 in winter. Taking the whole period, it is evident that small-pox is most fatal in winter and summer and least fatal in spring. To ascertain how far these results might be depended on, I divided this period into two as before, and obtained somewhat different results; in 1840-49 the highest mortality occurred in winter, 30·0 per cent., the next in autumn, 25·5 per cent., and the lowest in spring, 21·0 per cent. In 1850-56 the highest mortality also happened in winter, viz., 29·7 per cent., the next in spring, 26 3 per cent., and the lowest in autumn, 19·4 per cent. To show how considerable this variation was, I put the percentages in opposition to one another. In spring, 1840-49, the mortality was 21·0 per cent.; 1850-56, 26·3 per cent.; in summer, 1840-49, 23·5 per cent.; 1850-56, 24·6 per cent.; in autumn, 1840-49, 25·5 per cent.; 1850-56, 19·4 per cent.; in winter, 1840-49, 30·0 per cent; 1850-56, 29·7 per cent. I will put these in a tabular form.

*Percentages of Deaths from Small-Pox.*

| Years. | Spring. | Summer. | Autumn. | Winter. |
|---|---|---|---|---|
| 1840-56 ........ | 22·9 .... | 23·9 .... | 23·3 .... | 29·9 |
| 1840-49 ........ | 21·0 .... | 23·5 .... | 25·5 .... | 30·0 |
| 1850-56 ........ | 26·3 .... | 24·6 .... | 19·4 .... | 29·7 |

The two periods agree therefore in this, viz., that the winter is the season during which the mortality from small-pox reaches its culminating point. The question then arises, on what do these differences depend? As before stated, I can only reply to this in respect to temperature, and that very briefly.

An examination of the temperatures for the two periods shows that, whilst the mean temperature for the first period, 1840-49, during the spring quarter, was 1·3° in excess of the average since 1771, that of 1850-56 was 0·5° less, having been the coldest series of spring quarters since 1789. In the summer quarters the mean difference was very slight, viz., 0·2° only; in autumn, the mean temperature for 1840-49 was 0·8° above the average, whilst that of 1850-56 was 0·9° below; in winter the mean of 1840-49 was 0·9° in excess, and of 1850-56 no less than 1·6°. We, therefore, see that with a series of warm springs there was a small comparative mortality from small-pox, and with a series of cold springs there was a large comparative mortality; with summers of nearly equal warmth the rate of death varied 1·1 per cent., the largest mortality having happened also in the cold seasons; in autumn the smallest comparative mortality was coincident with the lowest mean temperature, and in winter with the highest. The highest mortalities in each period were coincident with a temperature of 37·6° and 38·7°; the periods of lowest mortality with a temperature of 46·5° in 1840-49, and of 56·5° in 1850-56. These latter results must be reasoned on with some caution, as the disease prevailed to a far greater extent in the first than in the second period.

Measles. I select measles as next in order for consideration, because it is so placed in the Registrar-General's Returns, and also because in its mortality at the different seasons it is more closely allied to small-pox than to any other of the eruptive fevers.

In considering the rate of mortality from small-pox, I have pointed out that there are two periods of elevation and two of depression. Measles, however, presents, in a marked degree, but one period of elevation and one of depression. The highest mortality registered in the ten corresponding weeks of the years 1840-49 was 395, and in the seven weeks of the years 1850-56, 202; the lowest mortality in the ten corresponding weeks of the years 1840-49 was 159, and in seven corresponding weeks of 1850-56 was 81: so that the greatest average weekly mortality from measles was rather above double its minimum, both in 1840-49 and in 1850-56. The highest mortuary rate in 1840-49 occurred during the fifty-first week, and in 1850-56 during the forty-ninth week; and the period of greatest mortality in each case commenced rather suddenly at the forty-eighth, and fell almost as suddenly in the fourth week, that is to say, occurred in December and January. In 1840-49 the rate diminished regularly, with one or two exceptions, to the sixteenth week, and then rose gradually to the nineteenth and twentieth weeks, when it took a leap, and then remained about stationary till the forty-fourth and forty-fifth weeks, when it took another leap to make its final rise at the forty-eighth week. During the years 1850-56, when the disease proved much less fatal, its course was somewhat different; for, having made a sudden descent at the fifth week, it as suddenly rose again at the seventh, and continued high to the seventeenth week, when it again declined somewhat, to rise again at the twenty-fourth week, to fall very suddenly at the thirty-fourth, and to obtain its lowest point at the thirty-eighth week, and thence rise to its maximum at the forty-ninth week. The aggregate mortality from this disease in the years 1850-56 was 20,676, of which 13,096 were registered in the years 1840-49, and only 7580 in 1850-56. Of the 13,096 deaths in 1840-49, 2436 were registered in spring, 3134 in summer, 3699 in autumn, and 3827 in winter; and of the 7580 in the years 1850-56, 2043 were registered in spring, 2002 in summer, 1370 in autumn, and 2165 in winter; or in the following ratios for the whole period, 21·7 per cent. in spring, 24·9 per cent. in summer, 24·5 per cent. in autumn, and 28·9 per cent. in winter.

These statistics, therefore, show that measles, like small-pox, is most fatal in winter, next in summer, and least fatal in spring—that is to say, if the aggregate quarterly numbers of the seventeen years be taken as the data. When, however, we come to compare the mortality in the two periods, we find a difference in the results as compared with the whole period, but a difference which is met with in both these diseases. In 1840-49 the mortality from measles during the winter quarter was 29·2 per cent. of that for the whole year, and in 1850-56, 28·6 per cent.; from small-pox it was 30·0 and 29·7 per cent. respectively. In the summers of 1840-49 the percentage mortality of measles was 23·9, and of small-pox 23·5 per cent.; whilst in 1850-56 that of measles was 26·4, and of small-pox 24·6 per cent. In the autumnal quarters of 1840-49 the rate of death from measles was 28·3, and of small-pox 25·5; whilst in 1850-56 it was only 18·1 per cent. from measles, and only 19·4 per cent. from small-pox.

A similar coincidence happened in the spring quarters, for in 1840-49 the mortuary rate from measles was 18·6, and from small-pox 21·0 per cent., whilst in 1850-56 it was 26·9 from measles, and 26·3 per cent from small-pox. Now, it is quite evident that some causes must have been in operation to have produced these results; and, as pointed out in considering small-pox, the springs of 1840-49 were unusually warm, and those of 1850-56 unusually cold. The same was the case in the autumns; whilst the winters of both periods were warmer than usual, and especially those of 1850-56. The conclusions, therefore, which we draw from these data are similar to those which have been deduced in regard to small-pox; viz., that as, during the spring quarters in which the temperature was above the average, the comparative rate of mortality from measles was low, and as during the cold springs it was high, a series of warm springs produces a less mortality from measles than a series of cold springs; and also that a similar statement applies to the winter quarters: whilst, on the contrary, the series of warm autumns were coincident with a high rate of mortality, and the cold autumns with an unusually low rate of deaths from measles. The highest mortuary rate in 1840-49 was coincident with a mean temperature of 39·7°, and in 1850-56 with 39·9°; its lowest rate in 1840-49 with 47·9°, and in 1850-56 with 56·2°. The following tabular arrangement enables the percentages of mortality to be read at a glance:—

*Percentages of Deaths from Measles.*

| Years. | Spring. | Summer. | Autumn. | Winter. |
|---|---|---|---|---|
| 1840-56 | 21·7 | 24·9 | 24·5 | 28·9 |
| 1840-49 | 18·6 | 23·9 | 28·3 | 29·2 |
| 1850-56 | 26·9 | 26·4 | 18·1 | 28·6 |

SCARLATINA. The rate of death from scarlet fever, although it presents a greater range between its highest and lowest points, oscillates, in a given period, much less than either small-pox or measles, and the periods of elevation and depression are subject to less variation. In the years 1840-56, the total deaths from scarlatina were no fewer than 33,451, of which 18,582 occurred in the ten years, 1840-49, and 14,869 in 1850-56. In each of these, the disease presented one period of elevation and one of depresssion; in 1840-49, the former extended from the thirty-eighth to the forty-fifth week inclusive, and in 1850-56 from the fortieth to the forty-seventh week, lasting, in each case, for eight weeks. The period of depression in 1840-49 commenced at the tenth week, and ceased at the nineteenth; in 1850-56 it began at the eighth week, and terminated at the sixteenth: the former period embraced ten and the latter nine weeks. The smallest number of deaths registered in ten corresponding weeks of the years 1840-49 was 215, and in seven corresponding weeks in 1850-56 it was 173; these numbers were registered in the fourteenth week in 1840-49, and in the eleventh of 1850-56. The commencement of the depression, as well as the week of lowest mortality, occurred earlier in 1850-56 than in 1840-49. This, probably, arose from the months of March in 1850-56 having been colder than those of 1840-49, and accords with the observation I made in my former essay on scarlatina, viz., "that when the temperature of March is higher than that of the previous February by an amount greater than the ordinary excess, the comparative mortality will also be in excess; and when the temperature is below the comparative and monthly mean, the comparative mortality will also be below the mean." The week in which the disease reached its culminating point in 1840-49 was the forty-first, when it caused 605 deaths, or nearly three times the number which were registered when the disease was at its ebb; in 1850-56 it was most fatal in the forty-fourth week, when it caused 462 deaths, nearly a similar increase to that which happened in the former period.

The highest mortality in any one year, if the year be dated from the 1st of January, does not always occur at its end, as a number of deaths, when the disease is epidemic, smaller than has occurred at its highest period, may be larger than will happen in the following autumn. This took place in the first weeks of 1853 and 1855, when the mortality from this disease was 67 and 85 respectively, and the greatest number of deaths in the following autumn was 59 and 76. To estimate the effects of atmospheric vicissitudes on this or any other epidemic disease, we should start from the time at which the smallest mortality occurs, trace the disease onwards to its highest point, and follow it until it again reaches its lowest; always bearing in mind that the cause must precede the effect for some definite period.

Of the total mortality in the years 1840-56, viz., 33,451, 6042 deaths happened in the spring, 7910 in the summer, 11,706 in the autumn, and 7793 in the winter quarters; or in the following ratio, 18·0 per cent. in spring, 23·6 per cent. in summer, 35·2 per cent. in autumn, and 23·2 per cent. in winter. This disease is, therefore, by far more fatal in autumn than in any other season, and, as before shown, rages most furiously from the middle or end of September to the middle of November; the largest number of deaths having occurred in October. I put the percentages in a tabular form.

*Percentages of Deaths from Scarlet Fever.*

| Years. | Spring. | Summer. | Autumn. | Winter. |
|---|---|---|---|---|
| 1840-56 | 18·0 | 23·6 | 35·2 | 23·2 |
| 1840-49 | 17·7 | 24·8 | 35·6 | 21·9 |
| 1850-56 | 18·5 | 22·2 | 34·3 | 25·0 |

The variation in the rate of death in the quarters of the two periods under examination was much less than in either measles or small-pox. In the spring quarters of 1840-49, the rate was 17·7, and of 1850-56, 18·5 per cent.; in the summers of 1840-49 it was 24·8, and of 1850-56, 22·2 per cent.; in the autumns of 1840-49 it was 35·6, and of 1850-56, 34·3 per cent.; in the winters of 1840-49 it was 21·9 per cent., and of 1850-56, 25·0 per cent. From these data it is evident that the greatest mortality from scarlet fever occurs in autumn, and the smallest in spring. The variation in the rate of mortality was greatest in the winter quarters; for in the winters of 1840-49, out of each 100 deaths, 21·9 per cent. occurred, whilst in 1850-56 no less than 25·0 per cent. happened. A comparison with the temperature shows that this increased rate of death was coincident with a series of unusually warm winters, which is in accordance with the results deduced in my former papers.

The rate of death in the two periods was by no means alike, 1858 deaths having been registered in each year of 1840-49, and 2124 in each of the years 1850-56, being an excess of one-seventh. This has arisen from the disease having assumed an epidemic form at shorter intervals than usual.

Fever. The rate of death from fever is far more uniform than from any other disease under consideration, and is far less affected by seasonal changes, for it ranged during the period 1840-56, between 23·4 per cent. in the spring quarters, when it was lowest, and 27·6 in the autumnal quarters when it was highest.

In the years 1840-49, 19,620 deaths from fever were registered, or at the rate of 1,962 per annum, and in the years 1850-57, 16,657 deaths occurred, or in the ratio of 2,379 deaths in each year; so that this disease has proved far more fatal since 1850 than in the previous ten years. How far this depends on greater fatality of the disease, or on the greater frequency of its occurrence, I cannot state; but this, and many other problems will, it is hoped, be solved by the weekly return of sickness now prepared and published from the statistics obtained by the medical officers of health.

The highest mortality in ten corresponding weeks of the years 1840-49 was 469, and the lowest 313, presenting a difference of 156, or an increase on the minimum mortality of one half. The increase on the minimum mortality of small-pox was rather more than double; of measles twice and a half; and of scarlet-fever about twice and four-fifths. The largest number of deaths in ten corresponding weeks of the years 1840-49 took place in the thirty-eighth and fiftieth weeks, and in seven corresponding weeks of the years 1850-56, in the fortieth and forty-third weeks. In 1840-49, the period of highest mortality extended from the thirty-sixth to the fifty-second week; and in 1850-56, from the thirty-sixth to the fifty-first week; corresponding more accurately than in the other eruptive fevers. The smallest number of deaths in ten corresponding weeks of 1840-49, was 313; and in seven corresponding weeks of 1850-56, was 264; having occurred in the former period during the fourteenth week, and in the latter during the ninth week. There was no lengthened period of depression in either of these periods.

The following is the rate of death in the different quarters of the period 1840-56; in the spring quarters 23·4 per cent., in the summer quarters 23·7 per cent., in the autumnal quarters 27·5 per cent, and in the winter quarters 25·3 per cent. These averages vary in a very small degree from those obtained by a consideration of the divided periods 1840-49 and 1850-56; for in the spring quarters of 1840-49 the rate was 23·4 against 23·5 per cent. in 1850-56; in the summer quarters it was, for 1840-49, 23·5 against 23·8 per cent. in

1850-56; in the autumns of 1840-49, 27·5 against 27·7 in 1850-56; and in the winters of 1840-49, 25·6 against 25·0 in 1850-56. These results are strikingly shown in the following table:—

*Percentages of Deaths from Fever.*

| Years. | Spring. | Summer. | Autumn. | Winter. |
|---|---|---|---|---|
| 1840-56 ........ | 23·4 .... | 23·7 .... | 27·7 .... | 25·3 |
| 1840-49 ........ | 23·4 .... | 23·5 .... | 27·5 .... | 25·6 |
| 1850-56 ........ | 23·5 .... | 23·8 .... | 27·7 .... | 25·6 |

The numbers of the mortality from fever show that these causes inducing the fatality, if not the outbreak of the disease, act with moderate uniformity at the different seasons of the year, and also in each year; still they show that season does not exercise a marked influence, as the aggregate deaths in the autumns of 1850-56 were 10,016 against 8,490 in the springs of the same years. An examination of the mortality for each week of 1850-56, shows the correctness of these statements. In 1850 the lowest mortality, 25, was registered in the seventeenth week of the year, and the highest, 56, in the forty-sixth; in 1851 the lowest, 18, occurred in the twenty-first week, and the highest, 79, in the forty-third; in 1852, the lowest, 23, in the twenty-fifth week, and 62 in the forty-third week; in 1853, the lowest was 30 in the twenty-fifth week, and 64 in the fiftieth week; in 1854, the lowest, 31, occurred in the ninth week, and the highest, 62, in the forty-fourth week; in 1855 the lowest occurred in the seventeenth week, and the highest, 65, in the fifty-first week; and in 1856, the lowest, 32, was registered in the twentieth week, and the highest, 76, in the forty-fifth week. The mortality from the disease was unusual throughout the whole of the year 1856, and the fluctuations were very great.

Diarrhœa. Although a consideration of the mortality from diarrhœa does not constitute part of our subject, yet it is so closely allied to it, that I think an analysis of its seasonal variations will not prove unacceptable, especially as the numbers of deaths has so very considerably increased during the last ten years.

It appears that no fewer than 29,308 deaths have been registered as caused by diarrhœa during the period now under consideration, of which only 13,264 were registered in the ten years 1840-49, against 16,044 in the seven years 1850-56, or 100 deaths in 1850-56 against each 58 in 1840-49. The highest number of deaths were registered in the thirty-second week of the period 1840-49, and in the thirty-third in the period 1850-56; in the former the number was 812, in the latter 1,234: the lowest mortuary rate occurred in the fourteenth week of the period 1840-9, and in the eighteenth of 1850-56.

The period of epidemic diarrhœa in 1840-49, was limited to the fourteen weeks from the twenty-eighth to the forty-first, and in 1850-56 to the fifteen weeks from the twenty-eighth to the forty-second. The period of highest mortality was extended from the thirtieth to the thirty-seventh weeks in 1840-49, and the thirty-first to the thirty-seventh weeks in 1850-56. During an equal period, seven weeks (thirty-first to the thirty-seventh inclusive) in 1840-56, no fewer than 12,766 deaths were registered, or more than two-fifths of the aggregate mortality; of these 5,463 happened in 1840-49, and 7,303 in 1850-56. Allowing, then, for a few days delay in registering the deaths, we may say that diarrhœa is ordinarily epidemic from the beginning of July to the first week in October, and that it reaches its maximum during the last weeks in July, the whole of August, and the first ten or fourteen days in September. The mean temperature of these weeks is as follows:—In an average of thirty-eight years—in the thirty-first week, 62·2°; thirty-second week, 62·0°; thirty-third week, 61·4°; thirty-fourth week, 60·5°; thirty-fifth week, 59·6°; thirty-sixth week, 58·5°; thirty-seventh week, 57·4°. The temperature of the thirtieth week was 61·9°; of the twenty-ninth, 62·0°; of the twenty-eighth, 61·9°; and of the twenty-seventh week, 61·9°. It is therefore evident that a mean weekly temperature of 60·5° or above, is coincident with an increase in the mortality from diarrhœa, whilst a fall below that temperature induces a reduction in the mortality.

From these statements we may infer that summer and autumn are the quarters in which the chief mortality from diarrhœa occurs; and an examination of the period 1840-56 shows this, for no less than 81·6 per cent. of the total number of deaths occurred during these quarters. The percentages of the mortality from diarrhœa are singularly uniform during each season, and show the intimate relation existing between a high temperature and a high mortality. There is no doubt that diarrhœa does prevail in very cold weather to a considerable extent; but the mortality from it at that season is much less. In the whole period, 1840-56, 8·5 per cent. of the total mortality happened in spring, 41·9 per cent. in summer, 39·7 in autumn, and 9·9 in winter; or, in 1840-49, 8·0 per cent. in spring, 42·8 per cent. in summer. 40·2 in autumn, and 9·2 in winter; and in 1850-56, 8·7 per cent. in spring, 41·3 in summer, 39·4 in autumn, and 10·6 in winter. These results may be contrasted best in a tabular form.

*Percentages of Deaths from Diarrhœa.*

| Years. | Spring. | Summer. | Autumn. | Winter. |
|---|---|---|---|---|
| 1840-56 ........ | 8·5 .... | 41·9 .... | 39·7 .... | 9·9 |
| 1840-49 ........ | 8·0 .... | 42·8 .... | 40·2 .... | 9·2 |
| 1850-56 ........ | 8·7 .... | 41·3 .... | 39·4 .... | 10·6 |

The correspondence between the rate of deaths in spring and winter is, I must confess, far more close than I expected previously to making this investigation.

As the results may be better comprehended by being eliminated from the body of the paper, I present them in as few words as possible.

1. *Small-pox* presents two periods of depressed and two of elevated mortality; the first period of elevation occurring in January, and the second at the end of May and early in June, the former being the highest; the first period of depression being at the end of March or early in April, and the second in September, the latter being the lowest; and, therefore, small-pox is most fatal in winter, and next in summer, and least fatal in spring.

2. A series of cold springs is attended with comparatively a large mortality; and the period of highest mortality coincided during these years with a temperature of less than 40° Fahr., and the lowest of above 46° Fahr.

3. The average highest mortality, in seventeen corresponding weeks of 1840-56, was more than double the average lowest mortality.

4. *Measles* has only one period of highest, and one of lowest mortality, the former occurring in December, and the latter varying in different years; but the rate of death in spring is much smaller than in any other quarter.

5. The rate of death was greater in the series of spring and winter quarters which were below the average temperature, than in those which were above it.

6. As in small-pox, the average highest mortality from measles during seventeen corresponding weeks in 1840-56 was more than double the average lowest mortality.

7. *Scarlet fever* presents one period of highest and one of lowest rate of death, and this more markedly than either small-pox or measles; the greatest number of deaths occurring at the end of October or beginning of November, the lowest at the middle or end of March or early in April.

8. The average mortality is higher in warm springs than in cold.

9. The mean of the greatest number of deaths from scarlet fever in seventeen corresponding weeks of 1840-56 was about twice and four-fifths as large as the mean of the lowest number of deaths in seventeen corresponding weeks.

10. Although there is a period at which *fever* is more rife than any other—viz., from the thirty-sixth to the fifty-first week—yet there is not any particular short period at which it can be said to reach its culminating point.

11. The greatest rate of death occurs in autumn, and the smallest in spring; the difference between them being less than in any other eruptive fever.

12. The average maximum number of deaths in seventeen corresponding weeks in the years 1840-56 was not much more than one and a half that of the minimum.

13. The period of greatest mortality from *diarrhœa* is very definitely marked, occupying a period of about seven weeks, (from the thirty-first to the thirty-seventh,) during which more than two-fifths of the annual mortality takes place, the greatest rate of death extending from the last week of July to the first week in September.

14. That this period corresponds with a mean weekly temperature of 60° or above.

It must not be inferred from these conclusions that I assume these diseases to be fatal in proportion to the increased rate of death at the different seasons of the year; for we have no statistics as yet to show that the rate of death, as compared with the ratio of attacks, becomes larger at the periods when these diseases are most prevalent. In the case of measles, as death is produced by inflammatory diseases of the respiratory organs, it is more than probable that although the mortality is greater during winter than at any other period of the year, yet the number of attacks may be smaller. These, and many similar statistical inquiries into the natural history of epidemic and other diseases, will most probably be elucidated by a strict investigation of the Weekly Return of Sickness, now published by the Board of Health, from documents supplied by the Medical Officers of Health of this metropolis.

